# A study on color visual perception of museum exhibition space based on eye movement experiments

**DOI:** 10.3389/fpsyg.2024.1431161

**Published:** 2024-11-11

**Authors:** Linhui Hu, Jinxiao Li, Lidan Chen, Qian Shan, Yu Jin, Guangpei Ren

**Affiliations:** ^1^School of Art and Design, Guangdong University of Technology, Guangzhou, China; ^2^Faculty of Innovation and Design, City University of Macau, Macau, China

**Keywords:** museum exhibits, environmental color, perceptual evaluation, eye movement experiment, visual perception

## Abstract

Color is an essential element in the exhibition space of museums, influencing people’s visual experience. To quantitatively study the relationship between color and individual visual perception in museum exhibition spaces, the study used eye-tracking technology combined with a questionnaire, exploring the relationship between color and visual perception in the exhibition environments of history museums in terms of hue, saturation, and brightness of color. Taking the Ruijin Central Revolutionary Base in Jiangxi Province as a historical display space for research, the results show that when the saturation is 69–77%, or the hue is red (0°H), the exhibited. environments have stronger color attractiveness. The study demonstrated that the first fixation duration in eye movement data significantly negatively correlates with the subject’s personal preference, comfort, pleasure, and attractiveness. Researchers can use this as an evaluation parameter for visual perception of exhibition space.

## 1 Research review

### 1.1 Museum exhibits

There are many different types of museums in China, and their numbers continue to grow yearly. To promote the high-quality development of China’s museum industry, the Propaganda Department of the Central Committee of the Communist Party of China, the National Development and Reform Commission, the Ministry of Culture and Tourism of the People’s Republic of China, the State Administration of Cultural Heritage, and nine other departments issued the “Guiding Opinions on Promoting the Reform and Development of Museums” on 24 May 2021, proposing that, by 2035, the system of museums with Chinese characteristics will be more mature and standardized, with improved social functions. The goal is to establish China as a world museum power, contributing Chinese wisdom and Chinese programs to global museum development (Ministry of Culture and Tourism of the People’s Republic of China, 2022). According to the definition of a museum issued by the International Council of Museums (ICOM) in 2007, museums are non-profit permanent institutions open to the public, serving society and its development. They collect, preserve, research, interpret, and exhibit the tangible and intangible heritage of humanity and its environment for educational, research, and enjoyment purposes (Dubois, 2009). The exhibition space is the core space of the museum. Ma (1994), in the “Museum General Theory,” summarized the traditional sense of the museum exhibition. According to this conventional understanding, a museum exhibition involves a certain number of physical objects arranged in a specific space and time, based on a specific purpose and a cohesive narrative. The design purpose of museum exhibition space is to effectively transfer knowledge and information to visitors. The purpose of museum exhibition space design is to efficiently gather and organize information, while thoughtfully and scientifically arranging elements such as space planning, structural design, graphic design, color schemes, lighting, and other key components to create a functional and aesthetically pleasing environment (Li, 2013).

### 1.2 Environmental color

The environment contains both natural and humanistic contexts. In the natural environment, the color environment generally refers to the colors and their variations found in the natural landscape, including the colors of the sky, trees, flowers, bodies of water, and other natural elements (Lynch and Livingston, 1995). In contrast, the humanistic environment involves the design and matching of colors within various artificial settings, such as architecture, interior design, urban planning, and artwork (Miller, 1997; Linton, 1999). In the museum exhibition space, the color environment comprises the ceiling, walls, floor, lighting, display cases, and exhibits.

Studies have shown that more than 80% of the information humans obtain from the outside comes from vision. Color (Gegenfurtner and Rieger, 2000), brightness (Treisman and Gelade, 1980), contrast, and motion (Franconeri et al., 2004) are the most easily perceived visual features. These features have priority in visual perception because they quickly draw attention and provide important information about the environment, showing the importance of color. As early as 1959, Guilford and Smith (Guilford and Smith,1959) found that color affects mood and that brighter, more saturated color leads to greater feelings of pleasure. Successive studies have proven that color can affect moods, such as the study proving that cool colors are pleasant (Mehrabian, 1994). Küller et al. (2006) investigated the effect of color on psychological mood in the work environment, with highly saturated colors being associated with negative moods. Following this, Küller et al. (2009) compared the psychological and physiological effects of gray, red, and blue rooms, and it was found that red rooms increased brain arousal levels. This effect was particularly pronounced in introverted or depressed people. Colors not only affect mood but also have a physiological impact. Gerard (1958) showed that warmer colors cause an increase in blood pressure, a faster heart rate, and a corresponding effect on the breathing rate and the number of blinks, while cooler colors have the opposite effect.

### 1.3 Eye movement experiments applied to color environments

The eye movement experiment is a series of eye movement behaviors, such as fixation and sweep, produced by a visual perception stimulus, thus responding to changes in the viewer’s perceptual and cognitive needs (Fischer and Weber, 1993). Meanwhile, the change of colors in visual perceptual stimuli is an important factor affecting human eye movements (Alipour et al., 2019). Studies have been conducted to apply eye movement experiments to spatial colors, and it is widely believed that space colors affect people (Sokolova and Fernández-Caballero, 2015). Tuszyńska et al. (2020) found that pastel color spaces, when analyzed through eye movement experiments, reveal a series of eye movement behaviors, such as fixation and sweep. These behaviors are responses to visual perception stimuli and reflect changes in the viewer’s perceptual and cognitive needs (Fischer and Weber, 1993). Meanwhile, the change of colors in visual perceptual stimuli is an important factor affecting human eye movements (Alipour et al., 2019). Studies have been conducted to apply eye movement experiments to spatial colors, and it is widely believed that space colors affect people (Sokolova and Fernández-Caballero, 2015).

In their study of the emotional experience of color space, Tuszyńska et al. (2020) found that pastel color spaces were more attractive and evoked positive emotions and that subjects’ pupil diameters became smaller when they perceived negatively emotional color spaces. Lee et al. (2005) investigated the existence of a correlation between color preference and eye movement patterns. Multivariate analysis of variance (MANOVA) showed significant differences in total fixation duration and total fixation count, and subjects preferred red and orange colors for more extended duration fixation for the most and least preferred colors. Zhang et al. (2022) conducted a study in the subway environment, revealing that people’s visual perception is more comfortable when the saturation is 48–60%, the brightness is 52–68%, or when the hue is orange, yellow, or green. The study also demonstrated that the pupil restlessness index and saccades rate, both part of the eye movement index, were significantly and negatively correlated with the subject’s comfort.

Meanwhile, Ming-Chung Ho et al. (2015) found a relationship between personal preference for color and comfort; however, physiological feedback does not readily change with color preference or comfort. Feng and Hao (2024) investigated changes in comfort in urban scenes and found that comfort in most cases showed an inverse relationship with dynamic duration and that colored light resulted in more varied individual experiential differences than white light at acceptable luminance levels. In summary, color significantly impacts people’s emotions and physiology.

Most studies on the color environment in museums primarily focus on lighting settings (Knez, 2001), indicating a need to expand research on the color environment within these spaces. Therefore, further research should combine psychological and physiological methods to explore in depth the comprehensive influence mechanism of the color environment on the visitor. This article combines eye-tracking technology with subjective questionnaire evaluation methods to study the effects of the three attributes of spatial color (hue, saturation, and brightness) on the psychological and physiological aspects of human beings by taking the historical display space of the Ruijin Central Revolutionary Base in Jiangxi Province as the research object.

## 2 Research methodology

### 2.1 Research design

To ensure the accuracy of the experimental results, it is necessary to minimize the influencing factors. Changes in the color of the roof, walls, floor, lighting, display cases, and exhibits can increase the complexity of visual stimulation and interfere with the judgment of the color of the exhibition space. By keeping parts of it the same, it is possible to analyze more accurately the specific impact of the wall color on the viewer’s visual perception. In human visual perception, vertical surfaces (walls) are more attractive than horizontal surfaces (floor and ceiling), and people’s sight naturally tends to focus on the vertical surfaces in front of them (Durgin et al., 2010; Flusberg et al., 2023). The change in wall color will directly affect the visitor’s visual focus and overall feeling. In contrast, the color change of the ground and ceiling has a less direct visual impact on the visitor, and the color of the display cases is usually a secondary factor.

To sum up, offline research conducted in several comprehensive museums identified a group of simple and uniform decorations of the basic display group as the experimental object. The experiment focused on the Ruijin Central Revolutionary Base in Jiangxi Province as both the historical display space and the experimental object. To measure human physiological sensations, the experiment was conducted with an eye movement instrument, and the dependent variables were questionnaire data and eye movement data. The eye movement data included the total saccade count, first fixation duration, total fixation duration, average pupil size, and total fixation count.

#### 2.1.1 Eye movement index

##### 2.1.1.1 Total saccade count

Saccade is the rapid movement of the eyes from one point of fixation to another. This fast movement helps us to quickly localize and reposition our focus in a visual scene to access information at different locations. Total saccade count refers to the number of rapid hopping movements performed by the eyes in a given period or during a specific task. Various emotional states (e.g., anxiety, tension, and pleasure) affect the saccade frequency. For example, saccade frequency may increase in an anxious state (Martinez-Conde and Macknik, 2008).

##### 2.1.1.2 First fixation duration

The first fixation duration can reflect how easy or difficult it is for the subject to extract information. The longer the duration, the more difficult it is for the subject to obtain information and the more uncomfortable the subject feels (Ministry of Culture and Tourism of the People’s Republic of China, 2022).

##### 2.1.1.3 Total fixation duration

The total fixation duration index is important for studying the degree of subject preference (Ministry of Culture and Tourism of the People’s Republic of China, 2022). Observers tend to spend more time focusing on targets that interest them or that they consider important. Therefore, a longer total fixation duration indicates greater appeal of the target.

##### 2.1.1.4 Total fixation count

In visual search, the total number of fixation counts is related to the amount of information the observer must process and not the depth of that information. Regions with a high amount of information produce a higher number of fixation counts; the total fixation count also increases in areas of interest to the searcher (JK, 2003).

##### 2.1.1.5 Average pupil size

Changes in the average pupil size affect them in two ways. On the one hand, emotional changes can also affect the average pupil. For example, strong emotions, such as anger, fear, and excitement, can cause the average pupil to dilate (Partala and Surakka, 2003). On the other hand, when cognitive load increases, the average pupil size dilates because the brain needs more resources to process complex tasks (Kahneman and Beatty, 1966).

### 2.2 Participants

In this study, 33 undergraduate and graduate students majoring in design at Guangdong University of Technology were recruited to participate in the test, and the sample consisted of 15 men and 18 women. The mean age of the participants was 23.2 years (SD = 1.2), respectively. By selecting participants with the same level of education, the influence of subjects’ *a priori* knowledge in dynamic visual cognition was excluded. All participants had normal or corrected-to-normal vision (5.0 and above), were free of strabismus, color blindness, or color deficiency, were right-handed, and had never participated in a similar study. Before the experiment, participants were informed that their eye movement data would be recorded and analyzed in a scientific research project and that all data would be anonymized. All participants provided written informed consent, and approval was obtained from the Human Subjects Research and Ethics Committee of the Guangdong University of Technology.

### 2.3 Stimulus material

The study explored the effects of different color factors on human comfort and personal preferences through quantitative studies of color. The current description of color is usually expressed quantitatively using a color model, such as RGB mode, CMYK mode, lab mode, and HSB mode. The HSB mode (H: hue[0°–360°], S: saturation [0–100%], B: brightness [0–100%]) is based on the visual acceptance mechanism of the human eye to simulate the color, compared to other color modes, it is more in line with the visual perception of the human eye, and it is more reasonable to use HSB mode in the experiment.

**Figure 1:**
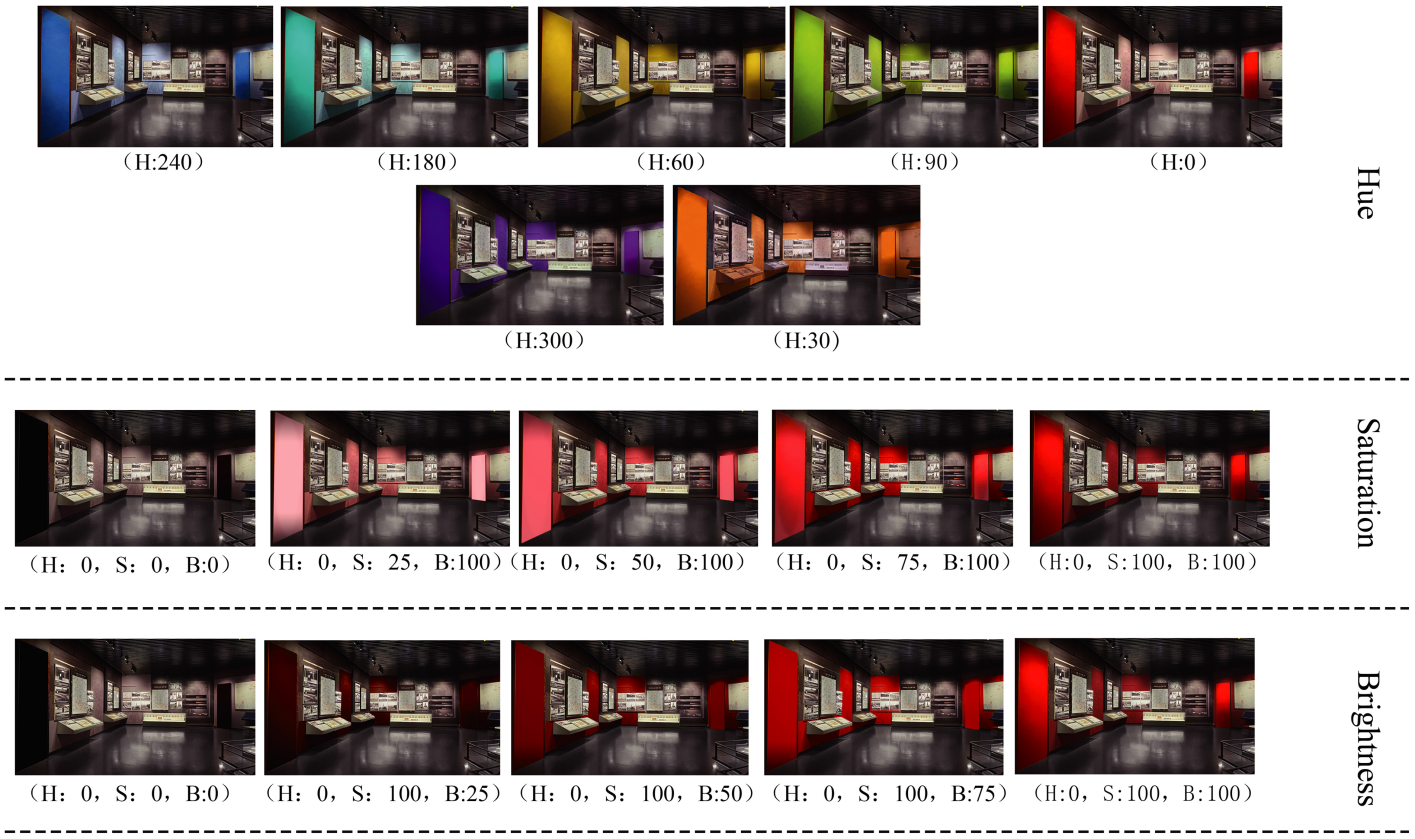
Hue, brightness, and saturation samples.

To ensure the selection of color samples was comprehensive and representative, we utilized the hue circle for color selection. There is no strict international standard regulation for hue division because the gradation of colors in hue is continuous rather than clearly segmented. In this process, we took 0° hue as the base point and chose hues every 30°. After discussions and consensus among the expert group, we screened out hues with minimal change. This resulted in seven representative colors: 0° for red, 30° for orange, 60° for yellow, 90° for green, 180° for cyan, 240° for blue, and 300° for violet. Some related studies have been found (Kalra and Karar, 2022; HWWE, 2019). If the saturation and brightness of all seven colors are tested, the subjects will feel fatigued; according to the Munsell system, the red color has the most obvious change in saturation, which can be divided into 14 levels, and the effect of changing saturation and brightness with it is the most obvious. The red color produces the most excitement and intensity of emotion. Therefore, we chose red as the representative color for the saturation and luminance experiment and extracted saturation and brightness at 0, 25, 50, 75, and 100% based on the red (0°) hue. The final sample is shown in Figure 1.

### 2.4 Experimental equipment

The instrument used in this experiment was a Tobil ProX3-120 eye tracker (sampling rate of 120 Hz, accuracy of 0.4), which records samples of eye movement data as subjects watch different scenes. The Tobil-Studio Professional software controlled the stimulus presentation, and stimulus images were presented on a 15.6-inch monitor (screen resolution 1920 × 1,080 pixels at 60 Hz). Participants sat 600–650 mm away from the centrally located monitor. All distractors (light, touch, noise, smell, etc.) were minimized during the experiment. The experiment was conducted in a 42-square-meter laboratory. Curtains were tightly closed, and artificial light was used to eliminate eyestrain. Eye movement data were recorded in real time using a human-machine synchronization system, the Ergo-LAB system (Version 3.7.3, Goldfarb Technology, China). The data were filtered and preprocessed for subsequent analysis.

### 2.5 Experimental procedure

First, the primary subject briefly introduced the experiment to the subjects and asked them to sign a consent form. The principal examiner adjusted the height of the subject’s chin rest and the height of the seat so that the subject’s eyes were roughly centered on the display screen. The subjects were also told not to move their heads as much as possible during the experiment to obtain high-quality data.

**Figure 2:**
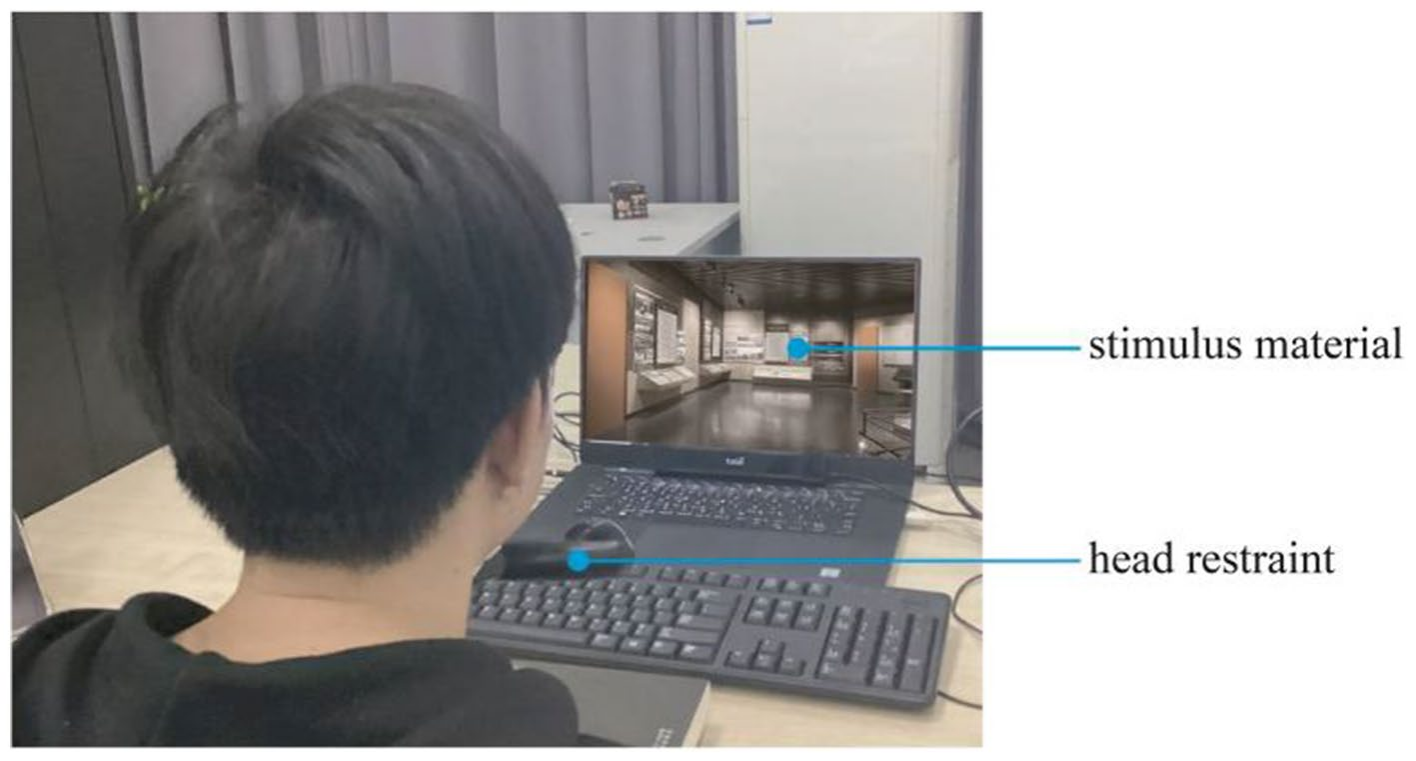
Experimental site.

The position of each participant’s eyes was calibrated before the images were displayed. This procedure required participants to look at a moving red dot on a white background and move their eyes to match the position of the red dot. The procedure was repeated once to verify calibration. After successful calibration, the stimulus was displayed on the screen, and eye movements were recorded (Figure 2).

**Figure 3:**
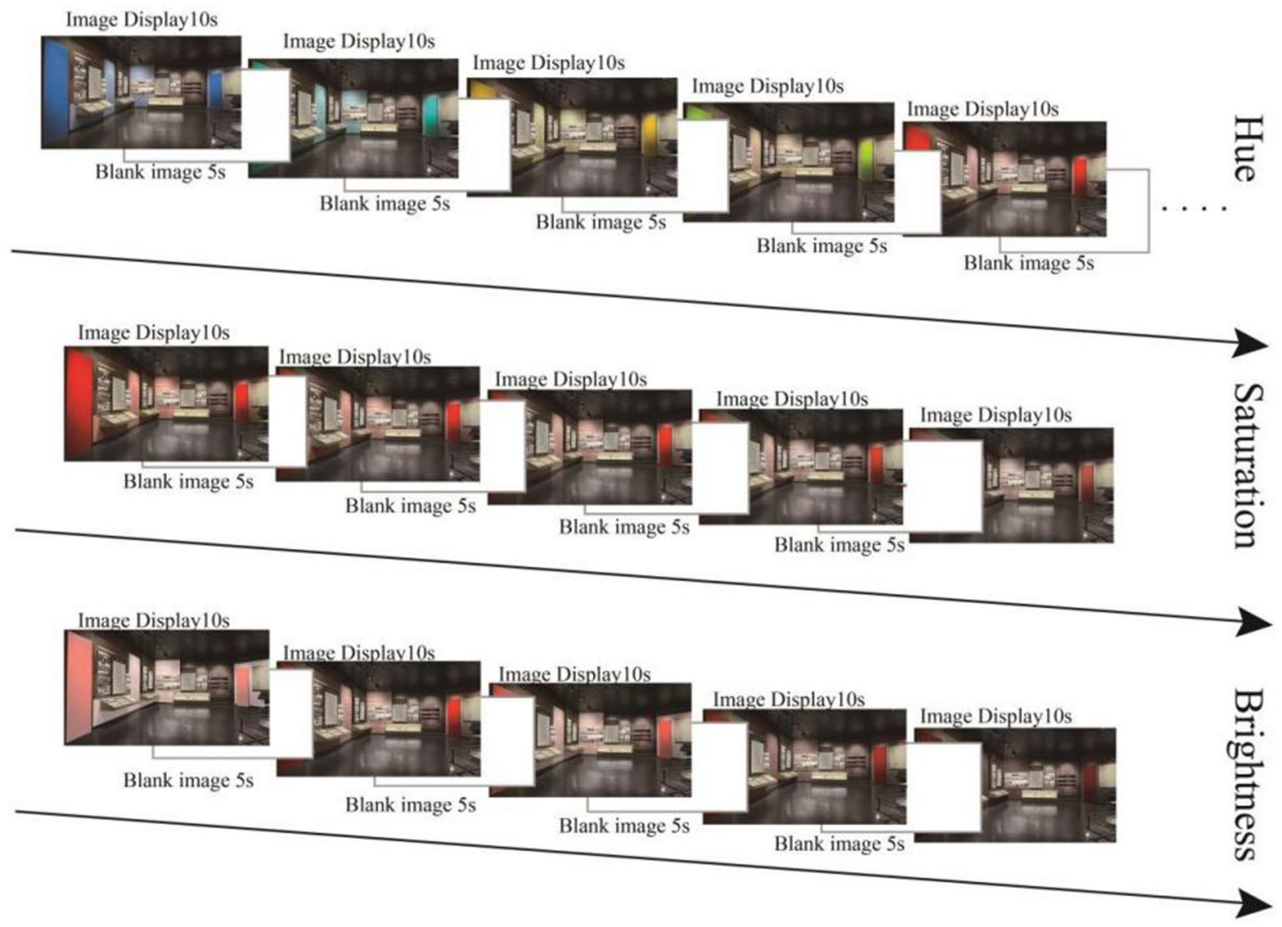
Hue, saturation, and brightness experimental procedures.

After the instrument was successfully calibrated, the experimental test was carried out. The test images were divided into three groups: the hue variable group, the brightness variable group, and the saturation variable group. A blank image with a central red dot popped up at the beginning of the experiment for 5 s. In each group of experiments, each test image was presented for 10 s. To eliminate the effect of the previous stimulus material on the measurement results, a blank image popped up at the end of each test image for 5 s. After each group of test images ended, the participants were given 15 s of relaxation time. To ensure more accurate data, the test images within each group were randomly presented on the screen; each image was presented one time, and the experiment totaled approximately 3 min and 25 s. At the end of the experiment, participants were asked to fill out a questionnaire about the subjective perception of scene viewing (Figure 3).

## 3 Results

### 3.1 Analysis of eye movement data

A total of 33 subjects were collected for a total of 561 sets of data, and among all the data, two subjects were excluded for their low sampling rate of eye movement data. The analysis of variance (ANOVA) of the eye movement data of 31 subjects was performed using the Spss27 software. The results show that under the hue variable, total saccade count (*F* = 13.536.68, *p* = 0.00), first fixation duration (*F* = 704.63, *p* = 0.00), total fixation duration (*F* = 304.7, *p* = 0.00), total fixation count (*F* = 87.37, *p* = 0.01), under the saturation variable, total saccade count (*F* = 3.147, *p* = 0.01), first fixation duration (*F* = 171.21, *p* = 0.01), total fixation duration (*F* = 347.55, *p* = 0.01), total fixation count (*F* = 19.61, *p* = 0.01) were all significantly different (*p* < 0.05).

However, under the influence of brightness, only the first fixation duration (*F* = 234.5, *p* = 0.01) showed significance. Other than this, none of the average pupil sizes showed significance.

#### 3.1.1 Total saccade count

**Figure 4:**
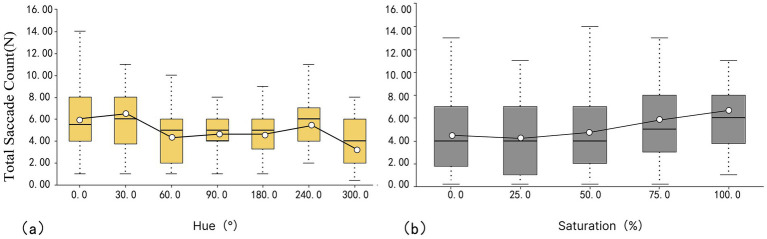
Box plot of total saccade counts: (a) hue and (b) saturation.

As shown in Figure 4A, the mean values from the hue box plot are arranged as follows: 300° < 60° < 90° < 180° < 240° < 0° < 30° (3.96 N < 4.63 N < 4.77 N < 4.82 N < 5.72 N < 5.93 N < 5.97 N). The lowest mean value corresponds to violet (300°), while red (0°), and orange (30°) exhibit higher mean values. This implies that purple has less mood variation, whereas red and orange have more mood variation. The green color (90°) exhibited the smallest IQR value of 2.000 ms, implying a small difference in the perception of visual fixation across subjects. In contrast, red and violet had the largest IQR value of 4.000 ms, implying a large difference in the perception of visual fixation across subjects.

The total saccade count for saturation as shown in Figure 4B, indicates mean values arranged as follows: 25 < 0 < 50 < 75 < 100% (4.08 N < 4.63 N < 5.35 N < 5.45 N < 5.97 N). The lowest mean value is associated with 25%, while the highest is for 100%. The IQR for 100% is small, at 4.25, implying that differences in visual fixation perception are smaller among the different subjects. In contrast, the IQR for 25% is larger, at 6, implying a large difference in visual fixation perception across subjects.

#### 3.1.2 First fixation duration

**Figure 5:**
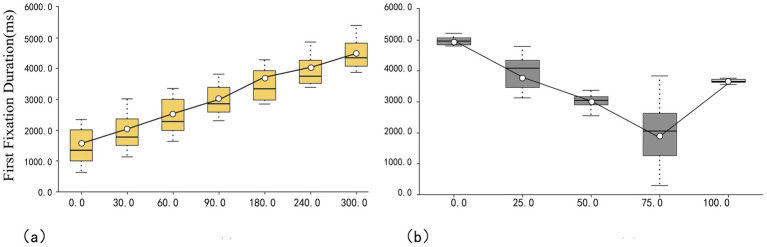
Box plot of first fixation duration: **(a)** hue and **(b)** saturation.

According to the data in Figure 5A, the mean values for different hues are as follows: 0° < 30° < 60° < 90° < 180° < 240° < 300° (1,438 ms < 1913 ms < 2,396 ms < 2,924 ms < 3,418 ms < 3,890 ms < 4,470 ms). The first fixation duration is longest for violet (300°) and shortest for red (0°), which implies that red is perceived as more attractive, while purple is seen as least attractive. In addition, 300°H has the smallest IQR value of 7376.5 ms, which means that the visual fixation difference between subjects is the smallest. In contrast, 0°H has the largest IQR value, which means that the visual fixation sensation difference between subjects is larger.

As shown in Figure 5B, the mean values for the first fixation duration for saturation are arranged as follows: 75 < 50 < 100 < 25 < 0% (2016 ms < 3,015 ms < 3,661 ms < 3,959 ms < 4,967 ms). The mean value for 75% saturation is the smallest, indicating that it is perceived as more attractive, while the mean value for 0% saturation is the largest, implying lower attractiveness. The smallest IQR value for 100%, at 997.5, implies the smallest difference in the perception of visual fixation across subjects. Conversely, the smallest IQR value for 75%, at 13,650, implies the largest difference in the perception of visual fixation across subjects.

#### 3.1.3 Total fixation duration

**Figure 6:**
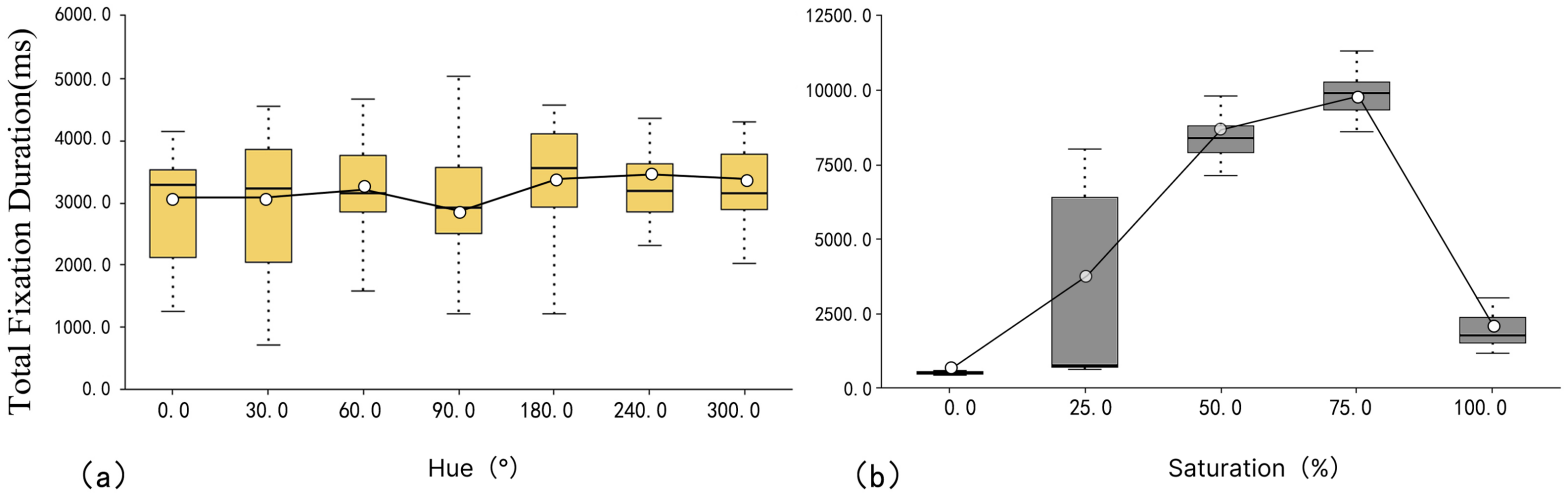
Box plot of total fixation duration: **(a)** hue and **(b)** saturation.

As shown in Figure 6A, the mean value of total fixation duration across different hues did not change significantly, with the following order: 30° < 90° < 0° < 300° < 60° < 180° < 240° (2,954 ms < 2,958 ms < 2,968 ms < 3,117 ms < 3,141 ms < 3,249 ms < 3,273 ms). The mean values of total fixation duration for orange (30°) and green (90°) were notably lower, while blue (240°), and cyan (180°) had higher values, implying that blue and cyan are more attractive, whereas orange and green are less attractive. Additionally, purple (300°) exhibited the smallest IQR value of 770 ms, reflecting the smallest visual fixation difference among subjects. In contrast, yellow (60°) has the largest IQR of 1829 ms, indicating significant variability in visual fixation among different subjects.

As shown in Figure 6B, the total fixation duration for saturation follows this order: 0 < 100 < 25 < 50 < 75% (490 ms < 1913 ms < 2,802 ms < 7,979 ms < 9,152 ms). The mean value for 0% saturation is the lowest, indicating that it is less attractive, while 75% saturation has the highest mean value, suggesting greater attractiveness. In terms of IQR values, 0% saturation has the smallest IQR at 868, implying the smallest difference in visual fixation across subjects. Conversely, 25% saturation has the largest IQR value at 56967.5, implying the largest difference in visual fixation across subjects.

#### 3.1.4 Total fixation count

**Figure 7:**
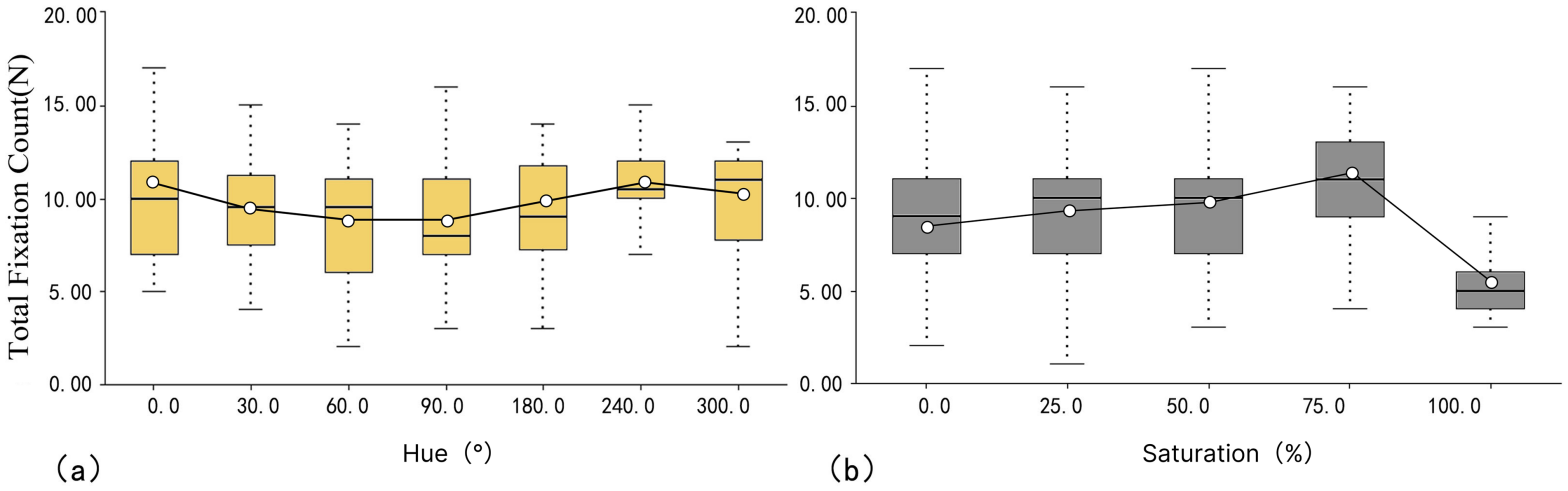
Box plot of total fixation count: (a) hue and (b) saturation.

According to Figure 7A, the mean value of total fixation count for hue is ordered as follows: 90° < 60° < 180° < 30° < 300° < 0° < 240° (8.37 N < 8.67 N < 9.00 N < 9.33 N < 9.54 N < 10.13 N < 10.6 N). The highest fixation count is found in cyan (240°), while the lowest is found in green (90°), indicating that cyan is the most attractive color and green is the least attractive color. Regarding IQR values, blue (240°) has the smallest IQR at 2.000, implying the smallest visual fixation difference among subjects. In contrast, red (0°) and yellow (60°) exhibit higher IQR values, implying greater variability in visual fixation among subjects.

As shown in Figure 7B, the mean fixation count for each saturation level is ordered as follows: 100 < 0 < 25 < 50 < 75% (5.25 N < 8.97 N < 9.32 N < 9.51 N < 11.01 N). 100 has the smallest saturation, and 75 has the largest saturation, which means that it is more attractiveness at the saturation level of 75%. The lowest attractiveness is found at the saturation level of 100%. In terms of IQR values, 100% has the smallest IQR at 2, suggesting minimal visual fixation differences across subjects. In contrast, all other saturation levels exhibit larger IQR values, indicating greater variability in visual fixation across subjects.

### 3.2 Questionnaire data feedback

**Figure 8:**
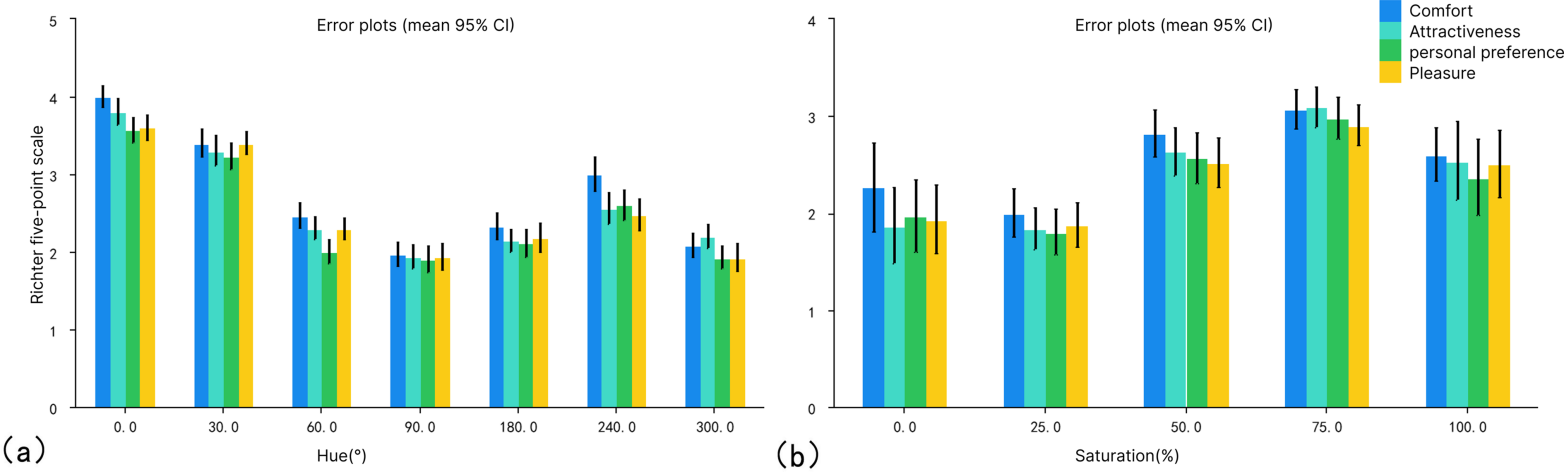
Subjective evaluation: (a) hue and (b) saturation.

A five-level Likert scale method was used to evaluate the comfort, personal preference, attractiveness, and pleasure of different color samples in the space. Comfort can be understood as physical sensations (e.g., temperature, humidity, and pressure) and psychological feelings (e.g., emotions, pressure, and sense of security). Personal preference refers to the degree of preference of a subject to a particular color environment. Attractiveness refers to the visual appeal of the color environment to the subject. This reflects the importance of the color environment in shaping first impressions and overall visual perception. The pleasure derived from a specific color environment contributes to visitors’ pleasure and satisfaction. Subjects rated the environmental color samples observed in the experiment by means of a subjective questionnaire on a scale of 5, with 1 being the worst visual perception and 5 being the best. Subjects’ subjective visual perception ratings were obtained by analyzing the questionnaire scores (Figure 8). It can be seen that the trend of color variables across different evaluation indices is consistent. The values for red (0°) and orange (30°) are higher than those of other colors, suggesting that the red–orange space may have a positive impact on subjects. Warmer colors have a better overall subject experience than cooler colors. The saturation variables show consistency across the different evaluation metrics, with all four metrics indicating that 75% saturation is higher than those for other saturation levels. This suggests that 75% saturation has a positive impact on subjects. Conversely, all four metrics for 25% saturation are lower than those for other saturation levels, indicating a negative impact on subjects.

## 4 Analyses

### 4.1 Correlation analysis

**Figure 9:**
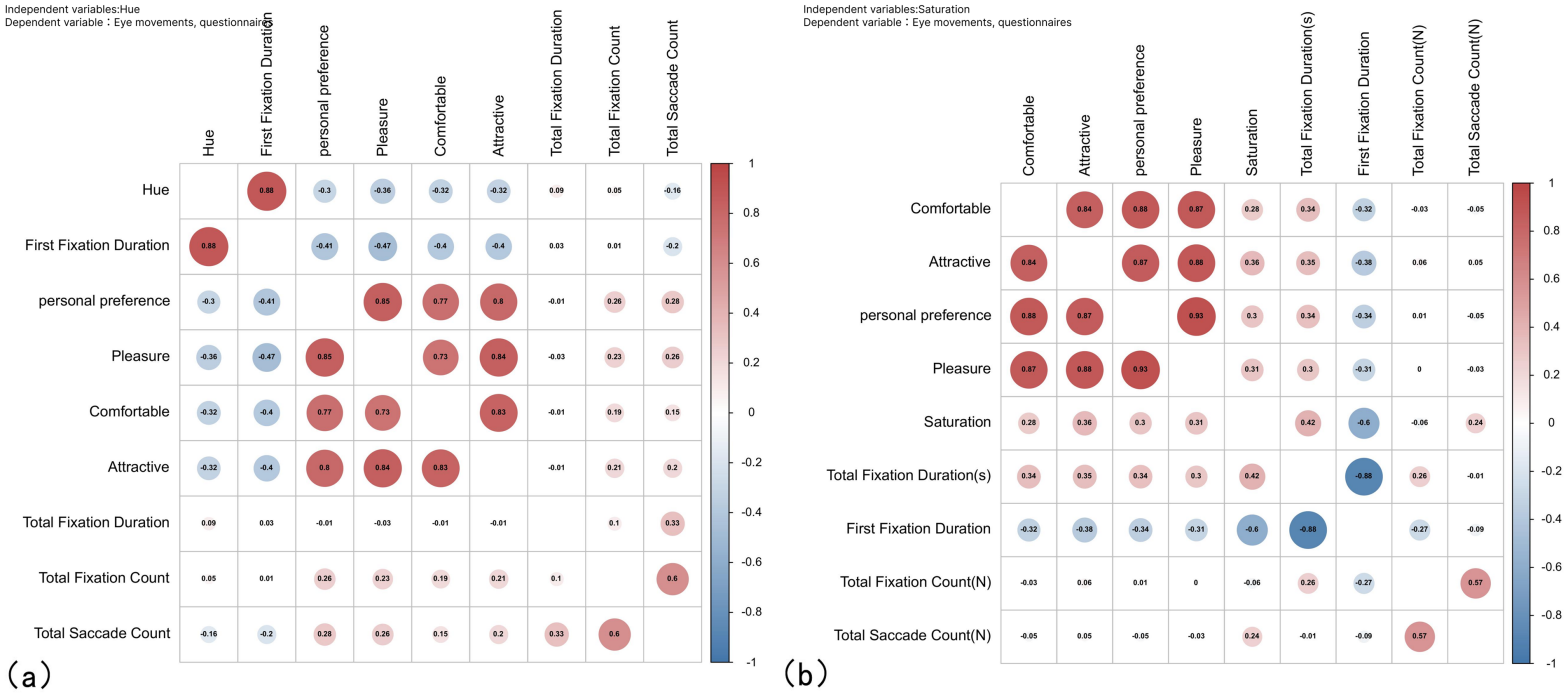
Correlation analysis of independent variables with dependent variables: (a) hue and (b) saturation.

Hue and saturation changes in color environments were found to have physiological and psychological effects on human beings. Correlations with eye movement indicators (first fixation duration, total fixation duration, total saccade count, and pleasure) and subjective questionnaires (comfort, attractiveness, personal preference, and pleasure), respectively, were analyzed to see the correlations (Figure 9).

There was a significant positive correlation between hue and first fixation duration (*r* = 0.884, *p* = 0.00), a weak positive correlation between hue and total saccade count (*r* = 0.112, *p* = 0.00), and a significant negative effect on the number of eye dips (*r* = −0.154, *p* = 0.01). There was no correlation between hue and total fixation count (*r* = 0.055, *p* = 0.085).

There were all significant negative correlations between hue and comfort (*r* = −0.321, *p* = 0.00), attractiveness (*r* = −0.300, *p* = 0.00), personal preference (*r* = −0.300, *p* = 0.00), and pleasure (*r* = −0.357, *p* = 0.00).

This was followed by a significant positive correlation of saturation on total fixation duration (*r* = 0.487, *p* = 0.01) and saccade count (*r* = 0.173, *p* = 0.01). Significantly negatively correlated with first fixation duration (*r* = −0.566, *p* = 0.01). Saturation was significantly and positively correlated with comfort (*r* = 0.264, *p* = 0.01), attractiveness (*r* = 0.339, *p* = 0.01), personal preference (*r* = 0.285, *p* = 0.01), and pleasure(*r* = 0.288, *p* = 0.01).

In addition, physiological indicators were found to correlate with subjective questionnaire evaluations.

Regarding hue, all four items of first fixation duration and comfort (*r* = −0.394, *p* = 0.000), attractiveness (*r* = −0.321, *p* = 0.000), personal preference (*r* = −0.412, *p* = 0.000), and pleasure (*r* = −0.473, *p* = 0.000) showed significance and negative correlation, which means that a shorter first fixation duration is associated with higher comfort, attractiveness, personal preference, and pleasure. Total saccade count had a positive correlation with comfort (*r* = 00.150, *p* = 0.000), attractiveness (*r* = 00.217, *p* = 0.000), personal preference (*r* = 0.284, *p* = 0.000), and pleasure (*r* = 00.261, *p* = 0.000), all of which were significant, suggesting that a greater number of eye skips is correlated with a higher level of comfort, attractiveness, personal preference, and pleasure. Total fixation count had a positive correlation with comfort (*r* = 00.184, *p* = 0.000), attractiveness (*r* = 00.213, *p* = 0.000), personal preference (*r* = 00.252, *p* = 0.000), and pleasure (*r* = 00.226, *p* = 0.000), all of which were significant. More fixation count was also associated with greater comfort, attractiveness, personal preference, and pleasure.

Regarding saturation, the total fixation duration showed a significant positive correlation with four items: comfort (*r* = 00.340, *p* > 0.05), attractiveness (*r* = 00.363, *p* > 0.05), personal preference (*r* = 00.303, *p* > 0.05), and pleasure (*r* = 00.316, *p* > 0.05). This indicates that as saturation increases, longer total fixation durations are associated with greater levels of comfort, attractiveness, personal preference, and pleasure. First fixation duration was negatively correlated with comfort (*r* = 0–0.326, *p* = 0.000), attractiveness (*r* = 0–0.377, *p* = 0.000), personal preference (*r* = 0–0.341, *p* = 0.000), and pleasure (*r* = 0–0.314, *p* = 0.000), all of which were significant, suggesting that when saturation is high, shorter first fixation duration is associated with higher levels of comfort, personal preference, and pleasure. Higher, shorter first fixation duration is associated with higher comfort, attractiveness, personal preference, and pleasure. Total fixation count and total saccade count were not significantly related to any of the four items of the questionnaire.

In terms of hue, the subjective evaluations of comfort, attractiveness, personal preference, and pleasure showed a significant correlation (*p* = 0.00). In the eye movement experimental index, the number of eye skips showed a significant positive correlation with total fixation duration (*r* = 00.328, *p* = 0.000) and total fixation count (*r* = 00.544, *p* = 0.000). Conversely, it exhibited a significant negative correlation with first fixation duration (*r* = 0–0.162, *p* = 0.000). This indicates a strong correlation between these subjective evaluations.

In terms of saturation, significant correlations were found among the four subjective evaluations of comfort, attractiveness, personal preference, and pleasure (*p* = 0.00), indicating a strong correlation between these subjective evaluations. Among the indicators of the eye movement experiment, total fixation duration count showed a significant positive correlation with total fixation duration (*r* = 00.261, *p* = 0.000) and saccade count (*r* = 00.579, *p* = 0.000) and a significant negative correlation with first fixation duration (*r* = 0–0.266, *p* = 0.000). First fixation duration negatively correlated with total fixation duration (*r* = 0–0.88, *p* = 0.000).

### 4.2 Curve fitting analysis

To further explore the relationship between color changes and physiological and psychological changes, the eye movement index and questionnaire index were used as the dependent variables, and color hue and saturation were used as the independent variables. Multiple linear regression analysis was performed on the independent variables and dependent variables, and the regression fitting curve was obtained.

**Figure 10:**
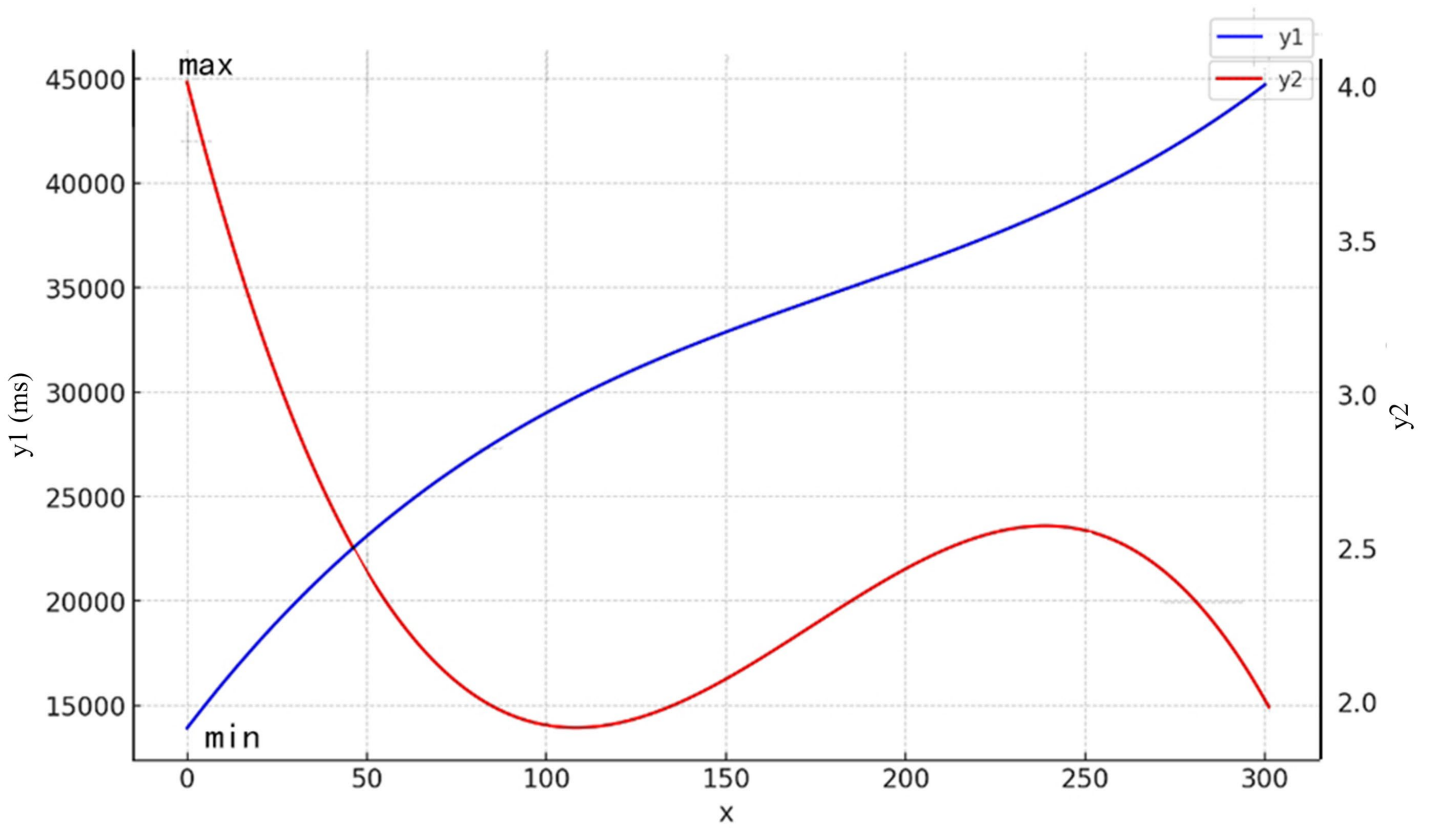
Curve fit: (x) hue, (y1) first fixation duration, and (y2) total fixation duration.

(1) The best fit was found for the hue versus first fixation duration cubic curve model (*r*^2^ = 0.807, *p* = 0.000), and the best fit was found for the hue versus attractiveness cubic curve model (*r*^2^ = 0.298, *p* = 0.000; Figure 10). To write the expression of the model based on the coefficients of the variables, the following format was used:

$$y=\beta_{0}+\beta x1+\beta_{2}x^{2}+\beta_{3}x^{3}$$

$$y_{1}=13926.38+224.94x-0.906x^{2}+0.001x^{3}$$

$$y_{2}=4.19-0.045x+0.0003x^{2}-5.95e^{-7}x^{3}$$

where $y_{1}$ is the first fixation duration score, $y_{2}$ is the attractiveness score, and (0 ≤ x ≤ 300) is hue. According to the analytical equation, y1 = 13926.39 and y2 = 4.19 reach their maximum values when x = 0. This means that attractiveness is strongest when the hue is red.

**Figure 11:**
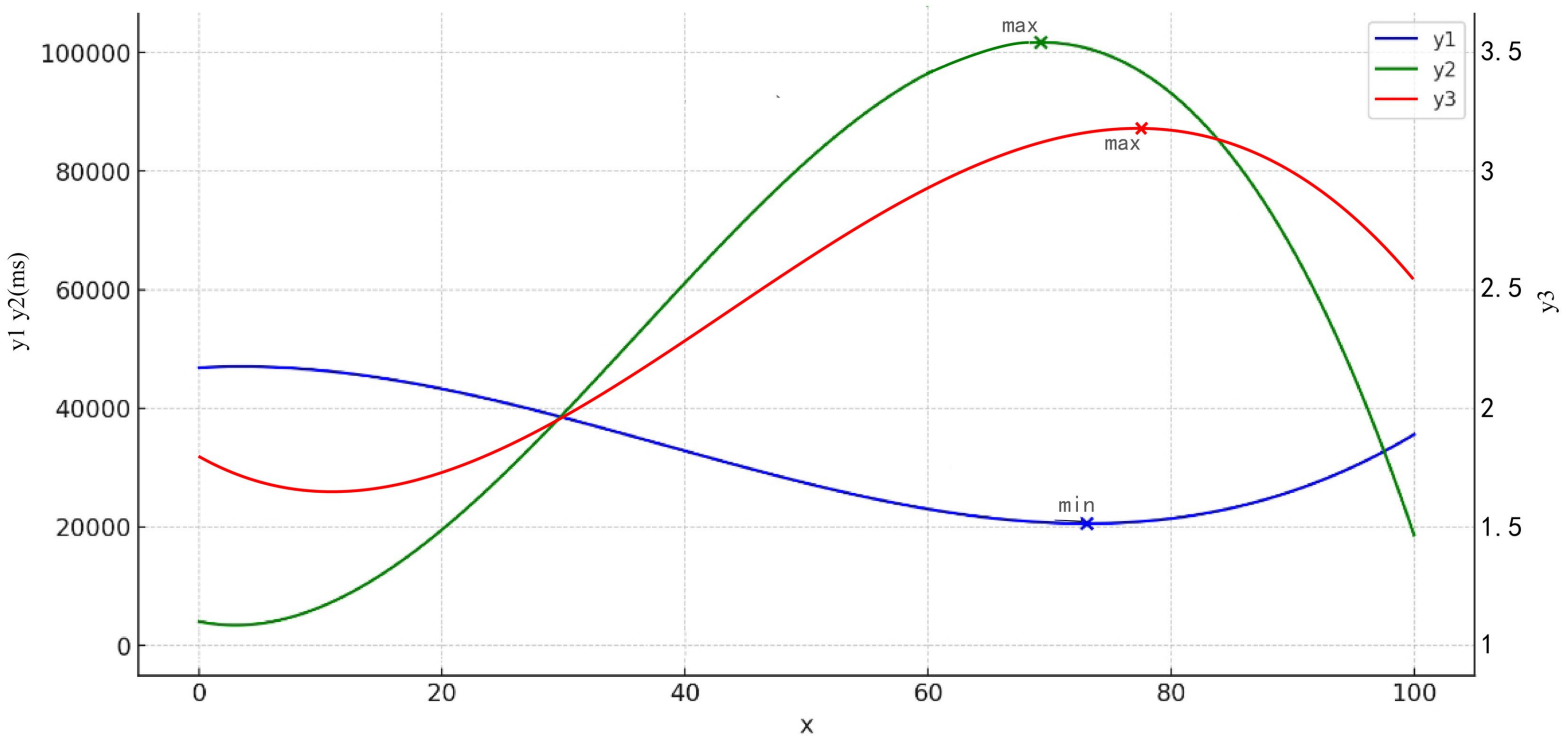
Curve fit: (x) saturation, (y1) first fixation duration, (y2) total fixation duration, and (y3) attractiveness.

(2) Saturation with first fixation duration (*r*^2^ = 0.753, *p* = 0.000), total fixation duration (*r*^2^ = 0.822, *p* = 0.000), attractiveness (*r*^2^ = 0.174, *p* = 0.000) were all best fitted with a cubic curve model (Figure 11). Based on the values of the coefficients of the variables, the expression of the model was written as follows:

$$y=\beta_{0}+\beta x1+\beta_{2}x^{2}+\beta_{3}x^{3}$$

$$y_{1}=46815.57+120.59x-18.08x^{2}+0.16x^{3}$$

$$y_{2}=4041.63-421.48x+73.24x^{2}-0.68x^{3}$$

$$y_{3}=1.85-0.03x+0.0001x^{2}-9.84e^{-\;6\;}x^{3}$$

where y1 is first fixation duration, y2 is total fixation duration, y3 is attractiveness, and x (0 ≤ x ≤ 100) is Saturation. According to the analytical equation, y1 = 20692.90 reaches its minimum value, when x = 73, and y2 = 101658.35 reaches its maximum value when x = 69. The maximum value of y3 = 3.07 is reached at x = 77, implying that the attractiveness is strongest when the saturation is between 69 and 77%.

## 5 Findings

This study utilized the basic display in the history exhibition hall as the research object. It adopted eye-tracking technology to obtain relevant data on subjects’ physiological indexes under different color attributes in the exhibition space. Through multivariate linear regression analysis, the interactions among hue, saturation, and subjects’ physiology and psychology responses in the exhibition space were revealed. It also explored the most suitable color design scheme for the exhibition space.

When the color hue of the museum space was red (0°), the subjects’ first fixation duration was lowest and more stable, and the subjects rated the space higher in attractiveness, personal preference, comfort, and pleasure. This result may be due to the influence of warm colors, which make people feel more active (Strandvall, 2009). and the effect of red in maintaining focus and improving attention to detail (Ou et al., 2004). Conversely, the visual perception of purple (300°) was poor; the first fixation duration was the longest, and the subjects rated the space lower in attractiveness, personal preference, comfort, and pleasure. This likely occurs because purple is often associated with mystery, depression, and melancholy (Bellantoni, 2012). In contrast, warm colors such as red are linked to pleasure and energy (Wang, 2022), making them more suitable for creating exhibition environments.

At a saturation level of 69–77%, subjects exhibit the shortest first fixation duration and the longest total fixation duration, indicating that their visual perception of attraction is at its strongest. The use of highly saturated colors, strong contrasting colors, and densely packed visual elements often causes visual fatigue or overload, making people feel uncomfortable (Glassman et al., 2016). Low-saturation colors may lack the ability to draw attention and stimulate emotions, making people feel emotionally uncomfortable (Strandvall, 2009).

In conjunction with the above discussion, both hue and saturation significantly affect the visual experience. Under the influence of hue and saturation, shorter first fixation duration and longer total fixation duration were generally associated with higher comfort, attractiveness, personal preference, and pleasure. Eye movement metrics (total saccade count and total fixation count) showed correlations across hue and saturation situations. In the case of hue, total saccade count and total fixation count had a significant positive correlation with subjective evaluation, whereas, in the case of saturation count and total fixation count, they had no significant relationship with subjective evaluation. The strong correlations between the subjective ratings (comfort, attractiveness, personal preference, and pleasure) indicate the consistency and interrelatedness of these metrics in assessing the visual experience.

In summary, this article investigated the relationship among color hue, saturation, and visual perception. Introducing objective physiological measurements into the study of spatial color allowed for the possibility of obtaining more accurate quantitative relationships. It also allowed for the optimization of the exhibition design and enhancement of the subjects’ experience.

## 6 Limitations

Studies have shown that populations in different countries can have different color preferences (Madden et al., 2000). The study population was all Chinese, and the color red has a special symbolic meaning for Chinese people (Zhang, 2018). People may subconsciously have a greater preference for the color red. The main research objective was to explain the relationship between individual spatial elements and human physiological indicators by controlling variables. However, there are many factors in the real environment, and the effects result from a combination of these factors. In the future, we will try to explore the interaction mechanism between multiple elements of space and human body indicators.

This article focuses exclusively on individuals aged 20–30 years and does not cover all age groups. Therefore, further research is needed to determine whether the conclusions can be applied to other age groups. In addition, spatial color may have different effects on different groups. Some existing studies on space have segmented the population based on gender and familiarity (Zhou et al., 2020; Kim et al., 2021). However, this study did not explore them in depth, which will be a direction for future research.

Our research was mainly conducted in the laboratory. While we have validated the models obtained in the laboratory using field questionnaires, some discrepancies with the real-world environments still exist. Therefore, establishing models that are based on the real environment will be a key focus for our future efforts.

## Data Availability

The raw data supporting the conclusions of this article will be made available by the authors, without undue reservation.

## References

[ref1] Alipour H. Namazi H. Azarnoush H. Jafari S. (2019). Fractal-based analysis of the influence of color tonality on human eye movements. Fractals 27:1950040. doi: 10.1142/s0218348x19500403

[ref2] Bellantoni P. (2012). If it's purple, someone's gonna die: The power of color in visual storytelling. New York, NY: Routledge.

[ref3] Dubois E. (2009). The structure and properties of color spaces and the representation of color images. Synthesis Lectures on Image, Video, and Multimedia Processing 4, 1–129. doi: 10.2200/s00224ed1v01y200910ivm011

[ref4] Durgin F. H. Li Z. Hajnal A. (2010). Slant perception in near space is categorically biased: evidence for a vertical tendency. Atten. Percept. Psychophysiol. 72, 1875–1889. doi: 10.3758/APP.72.7.1875, PMID: 20952785

[ref5] Feng K. Hao L. C. (2024). Dynamic lighting evaluation of media facades under visual comfort orientation - a comparative analysis based on laboratory and urban environments. Western J. Habitat 2, 144–150. doi: 10.13791/j.cnki.hsfwest.20240221

[ref6] Fischer B. Weber H. (1993). Express saccades and visual attention. Behav. Brain Sci. 16, 553–567. doi: 10.1017/s0140525x00031575

[ref7] Flusberg S. J. Shapiro D. Collister K. B. Thibodeau P. H. (2023). Things are looking up: vertical eye gaze in the environment affects perceptions of emotional valence in sad faces. Q. J. Exp. Psychol. 76, 1641–1657. doi: 10.1177/17470218221135429, PMID: 36250353

[ref8] Franconeri S. L. Simons D. J. Junge J. A. (2004). Searching for stimulus-driven shifts of attention. Psychon. Bull. Rev. 11, 876–881. doi: 10.3758/BF03196715 15732697

[ref9] Gegenfurtner K. R. Rieger J. (2000). Sensory and cognitive contributions of color to the recognition of natural scenes. Curr. Biol. 10, 805–808. doi: 10.1016/S0960-9822(00)00563-7, PMID: 10898985

[ref10] Gerard R. M. (1958). Differential effects of colored lights on psychophysiological functions (Doctoral dissertation, University of California, Los Angeles.).

[ref11] Glassman E. Guo P. Jackson D. Karger D. Kim J. (2016). 6.813/831 User Interface Design & Implementation. Available at: https://web.mit.edu/6.813/www/sp16/classes/16-color/ (Accessed July 1, 2024).

[ref12] Guilford J. P. Smith P. C. (1959). A system of color-preferences. Am. J. Psychol. 72, 487–502. doi: 10.2307/1419491 13830144

[ref13] Ho M. C. Chen J. M. Huang R. Y. Shen M. H. Lu M. C. Liu C. J. (2015). Numerical analysis on color preference and visual comfort from eye tracking technique. Math. Probl. Eng. 2015:861610, 1–4. doi: 10.1155/2015/861610

[ref14] HWWE (2019). Design science and innovation. Singapore: Springer.

[ref15] JK J. R. (2003). Eye tracking in human-computer interaction and usability research: ready to deliver the promises (section commentary). The mind's eye: cognitive and applied aspects of eye movement research. 41, 573–605. doi: 10.1016/B978-044451020-4/50031-1

[ref16] Kahneman D. Beatty J. (1966). Pupil diameter and load on memory. Science 154, 1583–1585. doi: 10.1126/science.154.3756.1583 5924930

[ref17] Kalra P. Karar V. (2022). “Effect of brightness on visual fatigue during video viewing” in Productivity with health, safety, and environment. eds. Singh L. P. Bhardwaj A. Iqbal R. Khanzode V.. J. Clin. Diagn. Res. 15:1.

[ref18] Kim J. Y. Choi J. K. Han H. Kim J. H. (2021). The influence of users’ spatial familiarity on their emotional perception of space and wayfinding movement patterns. Sensors 21:583. doi: 10.3390/s21082583, PMID: 33917017 PMC8067681

[ref19] Knez I. (2001). Effects of color of light on nonvisual psychological processes. J. Environ. Psychol. 21, 201–208. doi: 10.1006/jevp.2000.0198

[ref21] Küller R. Ballal S. Laike T. Mikellides B. Tonello G. (2006). The impact of light and color on psychological mood: a cross-cultural study of indoor work environments. Ergonomics 49, 1496–1507. doi: 10.1080/00140130600858142, PMID: 17050390

[ref22] Küller R. Mikellides B. Janssens J. (2009). Color, arousal, and performance—a comparison of three experiments. Color Research & Application: endorsed by inter-society color council, the color group (Great Britain), Canadian Society for Color, color science Association of Japan, Dutch Society for the Study of color, the Swedish color Centre Foundation, color Society of Australia. Centre Français de la Couleur 34, 141–152. doi: 10.1002/col.20476

[ref23] Lee T. R. Tang D. L. Tsai C. M. (2005). Exploring color preference through eye tracking. AIC color 3, 333–336.

[ref24] Li J. (2013). Research on color emotion in museum display design (Doctoral dissertation, Wuhan: Wuhan University of Technology).

[ref25] Linton H. (1999). Color in architecture: Design methods for buildings, interiors, and urban spaces. New York: McGraw-Hill.

[ref26] Lynch D. K. Livingston W. (1995). Color and light in nature. Cambridge: Cambridge University Press.

[ref27] Ma J. X. (1994). General introduction to museology: Sichuan University Press. doi: 10.4324/9780203520307

[ref28] Madden T. J. Hewett K. Roth M. S. (2000). Managing images in different cultures: a cross-national study of color meanings and preferences. J. Int. Mark. 8, 90–107. doi: 10.1509/jimk.8.4.90.19795

[ref29] Martinez-Conde S. Macknik S. L. (2008). Fixational eye movements across vertebrates: comparative dynamics, physiology, and perception. J. Vis. 8:28. doi: 10.1167/8.14.28 19146329

[ref30] Mehrabian A. (1994). Effects of color on emotions. J. Exp. Psychol. Gen. 123, 394–409. doi: 10.1037/0096-3445.123.4.394 7996122

[ref31] Miller M. C. (1997). Color for interior architecture. New York: Wiley.

[ref32] Ministry of Culture and Tourism of the People’s Republic of China. (2022). Circular of the general Office of the Ministry of culture and tourism, the general Office of the Ministry of culture and tourism, the general Office of the Ministry of education, and the Office of the State Administration of cultural heritage on the utilisation of cultural and tourism resources and cultural relics to enhance the spiritual literacy of young people.

[ref33] Ou L.-C. Luo M. R. Woodcock A. Wright A. (2004). A study of color emotion and color preference. Part I: color emotions for single colors. Color. Res. Appl. 29, 232–240. doi: 10.1002/col.20010

[ref34] Partala T. Surakka V. (2003). Pupil size variation as an indication of affective processing. Int. J. Human-Computer Stud. 59, 185–198. doi: 10.1016/S1071-5819(03)00017-X

[ref35] Sokolova M. V. Fernández-Caballero A. (2015). A review on the role of color and light in affective computing. Appl. Sci. 5, 275–293. doi: 10.3390/app5030275

[ref36] Strandvall T. (2009). “Eye tracking in human-computer interaction and usability research.” In *Human-computer interaction–INTERACT 2009: 12th IFIP TC 13 international conference, Uppsala, Sweden*, *august 24–28, 2009, proceedings, part II 12* (pp. 936–937). Springer Berlin Heidelberg.

[ref37] Treisman A. M. Gelade G. (1980). A feature-integration theory of attention. Cogn. Psychol. 12, 97–136. doi: 10.1016/0010-0285(80)90005-5 7351125

[ref38] Tuszyńska-Bogucka W. Kwiatkowski B. Chmielewska M. Dzieńkowski M. Kocki W. Pełka J.. (2020). The effects of interior design on wellness–eye tracking analysis in determining emotional experience of architectural space. A survey on a group of volunteers from the Lublin region, Eastern Poland. Ann. Agricul. Environ. Med. 27, 113–122. doi: 10.26444/aaem/106233, PMID: 32208589

[ref39] Wang X. (2022). Thinking about the construction of social mental service system and the development of PROFESSIONAL social work. Psychiatr. Danub. 34:414.

[ref40] Zhang Y. (2018). The gift of exchange: the name and reality of "Chinese red" under the perspective of art anthropology. Ethnic Art 2, 91–100.

[ref41] Zhang L. Li X. Li C. Zhang T. (2022). Research on visual comfort of color environment based on the eye-tracking method in subway space. J. Building Eng. 59:105138. doi: 10.1016/j.jobe.2022.105138

[ref42] Zhou Y. Cheng X. Zhu L. Qin T. Dong W. Liu J. (2020). How does gender affect indoor wayfinding under time pressure? Cartogr. Geogr. Inf. Sci. 47, 367–380. doi: 10.1080/15230406.2020.1760940

